# The Construct of Job Insecurity at Multiple Levels: Implications for Its Conceptualization and Theory Development

**DOI:** 10.3390/ijerph20043052

**Published:** 2023-02-09

**Authors:** Beatriz Sora, Thomas Höge, Amparo Caballer, José Maria Peiró

**Affiliations:** 1 Department of Psychology, University Rovira i Virgili, 43007 Tarragona, Spain; 2 Department of Psychology, University of Innsbruck, A-6020 Innsbruck, Austria; 3 Faculty of Psychology, University of Valencia, 46010 Valencia, Spain; 4 Faculty of Psychology, Universitat de Valencia & IVIE, 46010 Valencia, Spain

**Keywords:** job insecurity, job insecurity climate, climate strength job instability in organization, multilevel perspective

## Abstract

Over the years, job insecurity has accumulated important scholarly work. As a result, research has identified multiple constructs that involve employees’ concerns about job loss. Most of these are individual-level constructs (e.g., subjective and objective job insecurity), but, recently, an incipient body of literature has adopted a multilevel perspective by understanding job insecurity as a collective phenomenon (e.g., job insecurity climate, strength climate, downsizing or temporary hiring strategies). Furthermore, these constructs at different levels are underpinned by shared theoretical frameworks, such as stress theory or psychological contract theory. However, all this literature fails to present an integrative framework that contains the functional relationship for mapping job insecurity constructs across levels. Accordingly, the present study aims to examine job insecurity from a multilevel perspective, specifically by conceptualizing job insecurity at the individual level—understood as subjective and objective job insecurity—and at the organizational level, understood as job instability in an organization, job insecurity climate, and climate strength. The methodology of multilevel construct validation proposed by Chen, Mathieu and Bliese (2005) was applied; thus, (1) job insecurity were defined at each relevant level of analysis; (2) its nature and structure was specified at higher levels of analysis; (3) psychometric properties were tested across and/or at different levels of analysis; (4) the extent to which job insecurity varies between levels of analysis was estimated; and (5) the function of job insecurity was tested across different levels of analysis. The results showed significant relationships among these, and were related to an organizational antecedent (e.g., organization nature) and organizational and individual outcomes (collective and individual job satisfaction) in two European samples: Austria and Spain. Accordingly, this study exposed the multilevel validity of job insecurity constructs through an integrative framework in order to advance in the area of job insecurity theory and practice. The contributions and implications to job insecurity research and other multilevel research are discussed.

## 1. The Construct of Job Insecurity at Multiple Levels: Implications for Its Conceptualization and Theory Development

An important body of research has focused on studying job insecurity and related constructs involving job uncertainty, such as downsizing processes, job insecurity climate, subjective job insecurity, or temporary employment. Despite widespread attention from scholars, professionals and policymakers, several challenges remain to be addressed. For example, most of this research has adopted an individual perspective, overlooking the influence of contextual factors. Only recently, an emerging body of research has begun to examine job insecurity as a contextual construct from a multilevel perspective [1,2,3,4,5,6,7] (e.g., De Cuyper, Sora, De Witte, Caballer, and Peiró, 2009). In fact, the need to examine job insecurity at higher levels had already been stressed by Mohr [8], whose four-phase model hinted at the adoption of a multilevel perspective as an alternative path for research into job insecurity. This multilevel research is necessary because, according to Klein, Tosi and Cannella, “*Multilevel theories, thus, begin to bridge the micro-macro divide, integrating the micro domain’s focus on individuals and groups with the macro domain’s focus on organizations, environment, and strategy. The result is a deeper, richer portrait of organizational life—one that acknowledges the influence of the organizational context on individuals’ actions and perceptions and the influence of individuals’ actions and perceptions on the organizational context*” (1999, p. 243) [9]. The multilevel approach, therefore, has provided a set of benefits for research into job insecurity, and the research has significantly advanced.

However, there is a perceptible lack of a substantive framework for the validation of psychological constructs at multiple levels of analysis. Organizational researchers are likely to focus on either a micro- or macro-perspective, finding it difficult to design integrating theories [10]. It is necessary to understand the nature of constructs at higher levels before pursuing multilevel research and theory [11], as underlined by Chen, Mathieu and Bliese (2005) [12], who pointed out that multilevel construct validation is a fundamental aspect of advancing theory and practice in the areas of industrial-organizational psychology, organizational behavior, human resources, strategic management, and other related fields.

Where constructs depart from different levels (i.e., individual and collective levels), it is necessary to define the constructs in relation to these different levels and determine the characteristics they have in common. Differences of conceptualization in terms of level of analysis mean that, though these constructs may share similarities, they also manifest themselves in different ways. Collective constructs can be closely linked to their lower-level namesake, while differing in subtle and important ways [13]. For example, according to Schwartz (1994, p. 820) [14]: “the construct referenced on the ecological level [the group mean] may be the context or social environment in which individuals live, distinct from the attributes of those individuals. Thus, poverty as an individual characteristic and poverty as a neighborhood characteristic may exert different, independent effects on health”.

As far as we know, no study attempts to provide an empirically tested, multilevel definition of job insecurity according to a specific model, which determines whether processes and relationships among variables at the individual level are consistent with analogous processes and relationships at higher levels, such as the organizational level. In this study, we focus on the following constructs reflecting job uncertainties at the individual and the organizational level: objective job insecurity, subjective job insecurity, job insecurity climate, job insecurity climate strength, and job instability in organizations. Additional topics of importance may exist, but we believe these to be the core constructs of current job insecurity research.

To carry out our objective, we follow the principles of multilevel construct validation proposed by Chen, Mathieu and Bliese (2005) [12]. Thus, five consecutive steps were performed for the validation of job insecurity constructs at multiple levels: (1) define the focal construct at each relevant level of analysis; (2) specify the nature and structure of the construct at higher levels of analysis; (3) test the psychometric properties of the construct across and/or at different levels of analysis; (4) estimate the extent to which the construct varies between levels of analysis; and (5) test the function of the focal construct across different levels of analysis. The present article is organized according to the five phases of this validation process. However, validation of the meaningfulness of a construct at multiple levels involves, not only theoretical development, but also empirical testing, which is specifically required for steps 3, 4 and 5. A methodology section is therefore presented first, to explain how the empirical study was conducted. We conclude with a discussion of the implications for future research.

**Step 1. Construct definition: Conceptualization of job insecurity at multiple levels**

In defining the construct of job insecurity at multiple levels, we have deliberately focused on two of the most explored levels of theory and analysis in the multilevel organizational literature: individuals and organizations [9].

*Job Insecurity at Individual Level: An Individual Experience*

Individual job insecurity reflects the individual’s perceived threat of becoming unemployed. This concept has been defined in numerous ways in the literature. For example, Greenhalgh and Rosenblatt (1984, p. 438–4480) [15], defined job insecurity as “the perceived powerlessness to maintain the desired continuity in a threatened job situation”. Davy [16], understood job insecurity as employee “expectations about continuity in a job situation”, Van Vuuren and Klandermans [17] as “concern about the future permanence of the job”, and Sverke, Hellgren and Näswall [18] as the “subjectively experienced anticipation of a fundamental and involuntary event related to job loss”. Despite the differences, there is a common denominator in the content of these definitions. They all understand job insecurity as an overall concern about the continued existence of the job in the future [19], and they all agree on a set of aspects related to job insecurity. Firstly, job insecurity is a subjective experience or perception. The same situation can be perceived differently by different employees. Therefore, two employees in the same situation can have different levels of perceived job insecurity. Secondly, job insecurity involves uncertainty about the future. Employees who experience job insecurity are concerned about whether they will retain or lose their current jobs. The consequence of this is that they cannot adequately prepare themselves for the future because they are not sure what measures to take. This situation differs from that of certain dismissal, where employees are aware of their situation and can adopt the necessary measures to cope with the loss of employment. Thirdly, job insecurity is characterized by its involuntary nature and a feeling of powerlessness. The experience of job insecurity is not associated with a deliberate choice of an insecure job, but is related to a discrepancy between the level of job security preferred by the employee and that offered by the employer. Lastly, job insecurity involves feelings of helplessness in relation to maintaining job continuity. Although it is possible to find several conceptualizations of job insecurity that involve this involuntary, helplessness and uncontrollable nature, Shoss [20], in his critical review, suggested to exclude these aspects from job insecurity nature in order to clean up and clarify this phenomenon. Therefore, she recommended adopting a global construct of job insecurity that exists on a continuum from insecure to secure.

In contrast to the subjective definition of job insecurity, another line of research stresses that job insecurity is not generally a purely subjective, idiosyncratic perception, but rather is related to reality, and therefore to an objective, condition-related threat of job loss [21]. This threat may be produced by an imminent bankruptcy or the temporary nature of the job. This second possibility has been more extensively developed in the literature to increase understanding of the objective character of job insecurity [22]. According to the segmentation theory [23], the labor market and organizations can be divided into primary and secondary segments. The primary segment contains the primary jobs at the core of an organization, characterized by good working conditions and employment stability; whereas jobs belonging to the secondary segment are characterized by poorer conditions and employment instability. Temporary workers are positioned in the secondary segment because, compared to permanent workers (primary segment), they are not considered essential for the organization. Hence, the organization offers neither explicit nor implicit employment stability to temporary workers and, independently of individual perceptions, temporary employment is related to higher levels of job insecurity [22].

Other researchers differentiate between quantitative and qualitative job insecurity [24,25]. The quantitative perspective understands job insecurity as the perceived threat to the job itself; in other words, employees are uncertain about whether they will maintain their current jobs or become unemployed. The qualitative perspective refers to the continuity of valued aspects of the jobs, such as pay, working hours, colleagues, or job content. Finally, a cognitive and emotional differentiation has been drawn in the conceptualization of job insecurity. Cognitive job insecurity reflects the employee’s ideas and thoughts in relation to losing their job, compared to the emotional insecurity evoked by the feelings and fears associated with the cognition [26].

Despite the diversity in the conceptualization of job insecurity, there are two main paths of research into job insecurity at the individual level. One of these proposes that job insecurity is a subjective experience that involves concern and fear of job loss. The other refers to the idea that objective job insecurity is reflected by aspects of the employment relationship, such as temporary employment. Thus, job insecurity at the individual level has been mainly studied either as a subjective and quantitative phenomenon, or as an objective phenomenon, in terms of temporary employment [18,20,22,27,28]. Regarding subjective job insecurity, this study, following the study by Shoss [20], adopted a global construct of job insecurity.

Subjective job insecurity has mainly been studied from the stress theory perspective [29]. This theoretical framework defines job insecurity as a personal and individual experience, within which individuals appraise, first, the likelihood of job loss as a threat and, secondly, whether they have the necessary resources to cope with this threat successfully. Thus, the perception of job insecurity may vary across individuals in the same situation [30].

The consequences of objective job insecurity are also explained by a stress theoretical framework. It is argued that temporary employment involves more aggravating job characteristics [22,28]. This is congruent with assumptions from segmentation theory, as previously mentioned. More specifically, temporary employment implies specific stressors and impaired resources in four categories: job content (e.g., low autonomy, low skill utilization, role ambiguity), working conditions (e.g., painful and tiring positions, lack of information), employment conditions (e.g., poor wages, poor training and career development), and social relations at work (e.g., reduced involvement in participative decision-making processes) [28]. The nature and effects of objective job insecurity can also be derived from psychological contract theory. The psychological contract refers to the “perceptions about a set of mutual obligations that link employers and employees” [31]. Non-fulfillment of employee expectations on the part of the organization usually means a breach of the psychological contract [32]. To understand a psychological contract breach in the case of temporary and permanent workers, it is necessary to consider the breadth of the psychological contract, which varies according to the type of employment, with the psychological contract of temporary workers involving fewer expectations than that of permanent workers [33,34]. Temporary workers usually have a transactional psychological contract based primarily on economic exchange, whereas permanent workers have a relational psychological contract with an economic and a socio-emotional exchange. Hence, permanent workers are more likely to experience a stronger psychological contract breach than temporary workers because their psychological contract is more complex, with a greater number of expectations.

*Job Insecurity at Higher Levels: A Collective and Shared Experience*

There are a number of ways to understand job insecurity at higher levels. On the one hand, organizations are open systems with their own specific properties, characteristics, and structures [35]. These organizational factors shape a context in which individuals are exposed to common features, events, and procedures. A significant event involving job insecurity might be, for example, a downsizing process, whereby employees are exposed to a context of uncertainty and threat of job loss. In fact, a vast body of literature has demonstrated how organizational contexts characterized by job uncertainty and job instability due to downsizing can affect employees’ perceptions, attitudes and behaviors [31,36,37].

On the other hand, a certain degree of interdependence among employees in groups and organizations usually leads to bottom-up and top-down influence mechanisms [10]. Employees are nested in a similar context, in which some perceptions are more likely to develop than others. Individuals interact and share perceptions, which over time can converge into consensual views of the organization [38], leading collective perceptions to emerge from individual perceptions within organizations. This assumption underpins the basis of collective constructs such as organizational climate. Research has distinguished between the level of the organizational climate, understood as “the average of perceptions of employees within an organization” [39], and the strength of the organizational climate, conceptualized as “the degree of within-unit agreement among unit members’ climate perceptions” [40].

Against this background, we aimed to examine potential collective constructs related to job insecurity at the organizational level. More specifically, we examined job instability in an organization, understood as an organizational context in which an event or practice involving the reduction of a workforce has taken place. We also examined the emergence of job insecurity perception at higher levels: job insecurity climate and climate strength. Job instability could be seen as analogous to objective job insecurity at the organizational level, and job insecurity climate is analogous to subjective job insecurity at the organizational level.

*Job instability in an organization:* Throughout the most recent global economic crisis, an uncertain economic climate combined with the global marketplace has caused organizations to re-evaluate how they function. Many organizations have adopted measures to assure their survival and competitiveness. Mergers and acquisitions, restructuring, early retirement, or downsizing have become common practices, all of which involve the reduction of the workforce. In the aftermath of mergers and acquisitions, for example, personnel redundancies often arise. Employee downsizing represents a means by which the merged entity can remove slack and achieve operational synergies [41]. Downsizing is a managerial strategy that affects the size of the organization’s workforce and the work processes used [42,43]. Once a last-ditch measure to salvage a company, downsizing has become an accepted and almost routine management strategy [42].

Downsizing has an extraordinary impact on the organizational environment and its members. Appelbaum, Close and Klasa [44] pointed out that a downsizing process divides the workforce of an organization into two groups: one that leaves the organization, and another that remains. Confusion exists because employees who lose their jobs may not fully understand the reasons for their loss of employment; and those employees who remain may be perceived to have done little or nothing more to keep their positions. None of them can be said to truly responsible for their situations. Thus, the organization’s remaining workforce is likely to experience a “survivor syndrome”, defined as “the mixed bag of behaviours and emotions often exhibited by remaining employees following an organizational downsizing” [45]. In other words, while a downsizing operation is undoubtedly an emotionally wrenching process for the employees who lose their jobs, the survivors can have similar experiences [44,46]. Moreover, a downsizing process is often perceived as a violation of the psychological contract between employer and employees. Employees expect their contributions to the organization to be reciprocated with a stable and positive workplace. A downsizing process involves instability in the work environment, specifically with regard to the continuity of job positions in the organization. This idea is also underpinned by the stress theory perspective [29]. Although stress is understood as a subjective and personal experience, Lazarus and Folkman [29], emphasized that some events are generally perceived as stressful, and hence are deemed to be collective stressors. Lazarus and Folkman [29] also suggested that factors such as ambiguity and uncertainty are key in the perception of an event as stressful for most people. An environmental configuration is ambiguous when the information necessary for appraisal is unclear or insufficient. Uncertainty reflects the fact that people are confused about the meaning of the environmental configuration. Events that cause uncertainty can be stressful because they have an immobilizing effect on anticipatory coping processes. Both characteristics are present in a downsizing process, because employees rarely receive clear information and report concern about not knowing what is going to happen [47].

In this respect, empirical studies have shown how organizational downsizing creates an instable work environment, which is perceived as a threat by employees. More specifically, downsizing is experienced as a source of job uncertainty for employees, regardless of whether or not their own job is directly threatened [48,49]. Downsizing differs from job insecurity in its focus at the organizational level of analysis. According to this framework, job instability in an organization may be defined as a single generalized threat. In the present study, we define job instability in the organization as any organizational event that increases the possibility of job loss.

Several cross-sectional and longitudinal studies have also demonstrated that contexts characterized by job uncertainty have a harmful effect on employees, such as impaired mental and physical health, job attitudes or work behaviors [31,36,46].

*Job insecurity climate.* Organizational climate is defined as “the shared perception of the way things is around here. More precisely, climate is shared perceptions of organizational policies, practices and procedures” [50]. Conceptualized as a property at a higher level, organizational climate is generated as a result of the coalescence of individual perceptions, and hence focuses on how a situation is linked to worker perceptions [51].

Traditionally, the climate concept was used as a global term related to employee perceptions of different facets of the organization. However, this conceptualization was considered too amorphous, and more specific climates related to particular facets of the organization were proposed [52,53]. A single organization can have multiple climate types, according to the different facets of their environments [52], such as justice [54], safety [55], or affective tone [56]. Studies by Sora [5,6] showed that organizations also have a climate related to job insecurity, referred to as job insecurity climate. Sora [5] defined job insecurity climate as shared concerns about the continued existence of employee jobs. These authors lent empirical support to the construct of job insecurity climate with two different samples: a Spanish sample consisting of 20 organizations and 428 employees, and a Belgian sample consisting of 18 organizations and 550 employees. This study provided evidence of the emergence of collective concerns about job losses in organizations. The construct was replicated in subsequent studies conducted by the same researchers [5,6]. Later, Låstad [3,4], Jiang and Probst [57], Hsieh and Kao [1], Guidetti, Converso, DiFiore and Viotti [58], Tomas, Seršić, and De Witte, [59], and Nikolova [2] also provided additional evidence of the emergence of job insecurity climates as a higher-level construct in samples from Sweden, United States, Taiwan, Germany and Netherlands.

The emergence of job insecurity climates can be explained according to different theoretical frameworks. For example, following Schneider and Reichers(1983, p. 19–39) [53], organizational climates, generally speaking, emerge through three different processes: (1) a structuralist approach; (2) attraction–selection–attrition theory (Schneider, 1987); and (3) socialization theory.

The structuralist approach [60] suggests that the climate is generated by collective exposure to objective structural characteristics of the work context (i.e., size of the organization, centrality of decision-making, or the degree to which rules and policies constrain individual behavior). Members of an organization are subject to the same structural influences; hence they tend to experience similar perceptions. For example, in terms of job insecurity, human resource policies and practices may be essential to the emergence and nature of this climate. When the different dimensions of human resource policies and practices converge into carelessness and workforce instability as a result of, for example, poor labor conditions, temporality of contracts, lack of voice in the organization, lack of future or unclear career paths within an organization, it follows that employee perceptions will also converge in this direction. Furthermore, Schneider’s [53] attraction–selection–attrition theory proposes that organizations attract, select and retain employees with similar perceptions to those of the organization, as well as weed out those that deviate from those shared perceptions. Socialization theory [54] explains how old-timers transmit their perceptions, values and norms to newcomers, thus ensuring their assimilation into the organization.

Consistent with these models, other theoretical foundations may also contribute to explain how individuals can share their perceptions. For example, social comparison theory [61] states that individuals interact with referent others to better understand and make sense of uncertain situations, adapting their perceptions accordingly. Social information theory [62] also suggests that individuals use information drawn from their own past experiences and the thoughts of others to build their own perceptions. All these theoretical foundations support the assumption that individuals within a work context share their personal and individual perceptions, such as perceived job insecurity, leading to the emergence of a climate as a higher-level property (i.e., job insecurity climate).

Research further indicates that leadership is an important factor that shapes the formation and maintenance of climate perceptions [63,64]. Indeed, leaders have been labeled “climate engineers” [65].

According to stress theory [29], job insecurity climate can be understood as a collective job stressor. Stress may not just be an individual experience, but it can also present collective properties. From this perspective, the role of social groups, contexts, and intersubjective experiences of stress are highlighted in understanding the stress process [66]. Stress experiences can be shared by the members of an organization, leading to an organizational stress climate [67,68]. Apply to job insecurity, a job insecurity climate can be perceived as a threat (primary appraisal), and if the employees perceive that they do not have the necessary resources to cope with this threat (secondary appraisal), it can be a stressful experience. More specifically, the concern about the possibility of job loss is appraised and shared among employees within an organization through the different mechanisms (e.g., socialization, interaction, attraction–selection–attrition, etc.). This promotes a shared appraisal within organizations that this uncertainty is a collective threat. This process would represent the primary appraisal of stress, according to Lazarus and Folkman [29]. Later, and according to the secondary appraisal of stress, collective stressors cannot be merely coped with by individual strategies, but organizational coping strategies are necessary. In other words, individual efforts are not enough to cope with collective stressors, unless organizational-level initiatives are provided [69]. Hence, a generalized appraisal of not able to cope with the threat emerges. In sum, people can collectively generate a collective stress [67], or climate of stress [70]; thus, shared concern about potential job loss is perceived as stressful by the members of an organization. Finally, the climate of stress is a critical factor in explaining employees’ outcomes and may lead to strain [70]. In this vein, the job insecurity climate can be associated to detrimental outcomes. Sora [5,6] provided empirical support for this assumption by showing a negative relationship between job insecurity climate and employee work attitudes (e.g., job satisfaction and organizational commitment) above and beyond individual perceptions of job insecurity. Låstad [3] showed a negative relationship between job insecurity climate and health and a positive association with burnout. Hsieh and Kao [1] found a positive relationship between job insecurity climate and employees’ perceived organizational obstruction. Finally, Jiang and Probst [57] presented a negative association between job insecurity climate and safety outcomes.

*Climate strength*: Individuals tend to share their perceptions within organizations, leading to the emergence of constructs at higher levels such as organizational climate. Yet, individual differences persist within these organizations. Rather than being considered as error variance, this within-unit variance may be conceptualized as a focal construct in itself (i.e., climate strength). In relation to job insecurity, Sora, De Cuyper, Caballer, Peiró and De Witte [6] defined the construct of climate strength as “the degree to which employees of a specific organization agree on the level of perceived job insecurity”. A “strong” job insecurity climate reflects a high degree of agreement on perceived job insecurity within an organization, while a “weak” job insecurity climate is characterized by differences in perceived job insecurity among the members of a particular organization.

These differences within organizations can also be explained through different theoretical frameworks. First, the structuralist approach addresses the emergence of shared perceptions within work units, but also recognizes the variability in the degree of work-unit agreement. Although members of an organization are under the influence of the same human resources policies and practices, it is also obvious that these can be non-specific, or applied differently within the workforce, and that variability in the level of agreement about job insecurity perceptions can therefore arise. According to Mischel [71], situations present different degrees of ambiguity. In situations with little ambiguity, individuals are more likely to perceive events in a similar way, whereas ambiguous situations may lead to differences in perceptions of the same situation. Similarly, the interactive approach to climate formation (i.e., social information theory, social comparison theory) suggests that employees tend to interact in order to communicate and discuss their perceptions [53,72]. This explains how the interaction process facilitates shared interpretation, and this is reflected by the organizational climate (see above). With respect to the emergence of climate strength, it can be assumed that the greater the intensity of social interaction within an organization, the stronger the agreement of climate-relevant perceptions. Finally, regarding socialization theory and attraction–selection–attrition theory, Lindell and Brandt [73] reported that, in spite of employees with similar characteristics being selected and socialized to behave in a similar way, individual differences continued to exist within work units.

As for job insecurity and job insecurity climate, the basis for climate strength is stress theory [29]. However, the direction of the relationship varies among empirical studies. Some studies have suggested that low within-organization agreement is associated with detrimental outcomes [40,74,75,76]. A low level of agreement (i.e., weak climate strength) can be interpreted as a work stressor in organizations [40], liable to promote higher levels of conflict and stress compared to organizations with higher levels of agreement [73].

However, a number of studies have obtained contradictory results, and this is explained by the specific evaluative tone of the consensus [77]. A strong negative climate leads to detrimental reactions in employees, whereas a strong positive climate leads to positive reactions. Given that the strength of job insecurity climate reflects the degree of agreement about negative perceptions, Sora [6] found that a strong job insecurity climate was negatively related to employee outcomes (e.g., job satisfaction, organizational commitment and organizational trust).

**Step 2. Nature of job insecurity at multiple levels of analysis**

The nature of a construct represents the level of measurement needed to capture the manifestation of this construct. It is relatively straightforward to examine the nature of job insecurity at the individual level, given that the levels of theory, measurement and analysis are the same. This means that the researcher can draw conclusions and generalizations at the same level [10]. However, the nature of a collective construct is more complex, requiring the researcher to determine the type of aggregate-level measure that should be used to capture the higher-level manifestations of the construct [12]

Regarding job insecurity, we assume that, while the constructs reside at different levels, they are also related across these levels. As Morgeson and Hofmann [11] exposed, a construct may exist at multiple levels due to its *isomorphic* nature. In other words, a construct can have the same meaning across the different levels of analysis even when the manifestation of the construct differs across levels. This means that the nature of job insecurity constructs at higher levels is reflected by the specific *composition model* [78], which specifies the functional relationship between constructs at multiple levels of analysis with similar content. Chen, Mathieu and Bliese (2005, p. 380) [12] made the following observation about the role of the composition model: “the same multi-level construct can be manifested (i.e., measured) through different aggregated-level composition models, and so the particular composition model one uses should be based on one’s theory and research purpose, as well as practical considerations”.

Chan [78] proposed a typology of composition models to explain how lower-level data are used to establish constructs at higher levels. Although these forms represent the ideal types, the literature on work and organizational psychology has preferentially applied the direct consensus model to multilevel constructs; and, more recently, the dispersion model is beginning to attract attention. The present study conceptualized job insecurity climate according to the direct consensus model, and climate strength according to the dispersion model. Chen, Mathieu and Bliese [12] proposed an additional model: the aggregate properties model. Consistent with this model, we analyzed the construct job instability in the organization.

*Measures of job insecurity* vary across the studies in the literature according to the diversity of their definitions. The first measures of job insecurity we find in the literature are single items related to employee beliefs about retaining their current job in an unforeseeable future [79,80]. However, measures of job insecurity progressed as the literature began to expand, and a wide range of measurements can now be found. For example, objective job insecurity was operationalized through temporary work [19,22,81]; and subjective job insecurity was measured through diverse scales that assessed the quantitative dimension—the overall concern about potential job loss [19,82], or the qualitative dimension—that represents the threat of losing valued aspects of the current job [83]. Measures of cognitive and emotional job insecurity can also be found too, such as the scale developed by Pienaar [80]. Despite their differences, the referent of all these measurements and scales is the individual experience.

*Job insecurity climate* reflects employees’ shared concerns about the continuity of their jobs within a particular organization. This concept is isomorphic and hence analogous to job insecurity at the individual level. As explained by Bliese [13], “the aggregate maintains close links to its lower-level counterpart but nevertheless differs in subtle and important ways”. In other words, collective constructs, though qualitatively different in their essence, maintain conceptual links with their lower-level counterparts. Specifically, conceptualizations of job insecurity at the individual level and job insecurity climate at the organizational level both involve concern about job loss; however, the referent of job insecurity is the individual, whereas the referent of job insecurity climate is the collective concern of the members within an organization. We assume, therefore, that the measurement of job insecurity climate is based on a direct consensus model, and that, while job insecurity and job insecurity climate are analyzed with the same measures, they have different meanings at different levels. Operationally, job insecurity climate within an organization can be assessed by aggregating individual perceptions of job insecurity across members of the organization, with the average representing the climate level for the organization. The within-organization consensus, as indexed by agreement of lower-level perceptions, is used to compose the higher-level construct. High agreement at the lower, individual level establishes the basis for justifying the aggregation and evidencing variables at the higher, organizational levels [78], because it reflects *shared* perceptions and thus an emergent “climate”, as opposed to merely different idiosyncratic perceptions. In sum, consistent with Chan [78], the conceptual definition of job insecurity climate, together with the aggregate procedure and preconditions, determine the meaningfulness and validity of the operationalization of this construct at higher levels.

*Climate strength* is based on the notion of within-unit agreement, and consequently on the dispersion model. This model presents “the use of within-group dispersion (i.e., variance or agreement) to specify the functional relationship in composition of a dispersion construct and on the conceptual and methodological considerations” [78]. Unlike the climate concept, based on within-unit agreement, the foundation of the climate strength concept is within-unit variance. Climate strength reflects the dispersion of individuals’ perceptions in an organization, and is, therefore, an organization-level characteristic. In its operationalization, within-organization dispersion is simply the result of individual differences within the organization. Thus, job insecurity climate strength is indexed using within-organization variance or another dispersion measure of individual job insecurity response.

*Job instability in the organization* is understood using the aggregate properties model [12], in which the higher-level constructs are directly aligned with the higher-level unit. More specifically, the aggregate properties model represents index higher-level constructs directly aligned with the unit level of analysis. Thus, its measure is collected directly at the aggregate level (organizational level) instead of obtaining the measures from lower-level units or individuals. In this way, the level of the construct, its measurement, and its use in substantive analyses are all aligned [12]. In our case, the construct of job instability in the organization is usually measured directly at the organizational level. Job instability occurs in the aftermath of organizational events involving the reduction of the workforce. Job instability in the organization can be operationalized by ascertaining where and when such events have taken place, e.g., by asking employers about the size of the workforce in recent times, and inquiring about measures usually associated with job instability (e.g., downsizing, mergers, layoffs, etc.).

## 2. Method

### 2.1. Procedure

Samples were selected from two European countries, Spain and Austria, in order to provide major support to our results and to facilitate their extrapolation. These countries present different cultures. Cultural values are more strongly associated to one’s nation than to organization, individual personality or religion [84,85]. In fact, cultural values serve as “guiding principles in the life of a person or other social entity” [14] and they influence how individuals perceive, set goals and interpret relationships, expectations, demands, and duties in the workplace [86] Triandis (1996) proposed that individualism and collectivism values are important lenses through which to view psychological functioning [87]. Individualistic cultures focus on individual independence, individual goals, autonomy, and pay attention to the costs and benefits of relationships, while collectivist cultures emphasize the welfare of the group, the goals of the ingroup and define the self as interdependent [87]. Extended research has evidenced how cultural values affect work-related factors, such as work stress and job satisfaction. For example, Huang and Van de Vliert (2004) showed a link between job satisfaction and job status in individualist cultures, but not in collectivist ones [88]. Klassen [86] found that job stress was negatively associated to job satisfaction for North American teachers (individualist culture), and collectivism values were associated to job satisfaction for Korean teachers. In this sense, Spain presents a tendency towards collectivism and Austria towards individualism [89]. There are also differences between the two countries in terms of their economies and labor markets. The Austrian labor market is more stable with lower rates of unemployment and temporary employment compared to Spain. In fact, at the time of our investigation in 2011, Spain was more significantly affected by the world financial crisis. Among the countries of the European Union, Spain presented the highest and Austria the lowest unemployment rate. The unemployment rate in Spain in 2011 was about 21%—compared to about 4% in Austria [90].

In both countries, researchers sought the collaboration of the human resources departments of a number of organizations. The purpose of the research and main features of the questionnaire were explained, guaranteeing anonymity and confidentiality. Once organizations had agreed to collaborate, all their employees who wished to participate were invited to complete questionnaires, one type for the employees and another for the employers. The questionnaires were distributed at the workplaces. Most of employees and employers filled in the questionnaires in the moment of receiving them. Research assistants were present where participants filled in the questionnaires, and they could clarify any doubt or question. Those participants, who could not fill in the questionnaire at that moment, could fill in this later. More specifically, one week later, the research assistant came back to the workplace to collect these filled questionnaires. Only an employer per organization completed the questionnaire in representation of the organization. A wholly random sampling method was not possible, given the reliance on voluntary participation.

All subjects gave their informed consent for inclusion before they participated in the study. The study was conducted in accordance with the Declaration of Helsinki, and the protocol followed the guidelines of the Ethics Committee of our university.

### 2.2. Sample

The total sample was made up of 1435 employees from 138 organizations, selected from four labor sectors (construction, retail, health care and education), and two European countries, Spain and Austria.

The Spanish subsample was composed of 927 employees (65%) and 88 organizations (64%), with a construction sector component of 16 organizations (18%) and 136 employees (15%), a retail sector component of 31 organizations (35%) and 278 employees (30%), a health care sector component of 16 organizations (18%) and 205 employees (22%), and an education sector component of 25 organizations (28%) and 308 employees (33%). Eighteen of the organizations were public (21%) and 67 were private (76%); 584 of the employees were women (63%), and 317 were men (34%), with a mean age of 39 years (SD = 10.19). Permanent contracts were held by 78% of the employees (*n* = 724), and 21% had temporary contracts (*n* = 193).

The Austrian subsample consisted of 508 employees (35%) and 50 organizations (36%), with a construction sector industry composed of 10 organizations (20%) and 84 employees (16%), a retail sector component of 16 organizations (32%) and 165 employees (32%), a health care sector component of 11 organizations (22%) and 126 employees (25%), and an education sector component of 13 organizations (26%) and 133 employees (26%). Fifteen of the organizations were public (26%) and 30 were private (52%); 315 of the employees were women (62%) and 179 were men (35%), with a mean age of 37 years (SD = 11.56). Permanent contracts were held by 78% percent of the employees (*n* = 396), and 20% had temporary contracts (*n* = 102).

### 2.3. Measures

As previously mentioned, two different questionnaires were distributed, one for employees and another for employers. In the employee’s questionnaire, the following variables were measured: subjective and objective job insecurity and job satisfaction; whereas in the employer’s questionnaire, we measured job instability in the organization and nature of the organization.

*Subjective job insecurity* at the individual level was measured using a four-item scale proposed by De Witte (2000) [91]: (1) “It is very likely that I will soon lose my job”; (2) “I feel insecure about the future of my job”; (3) “I think I might lose my job in the near future”; (4) “I am sure I can keep my job”. The response options varied from 1 (“strongly disagree”) to 5 (“strongly agree”). Although the multilevel construct validation method [12] does not include the analysis of measurement invariance, we examinate this issue to clarify that there is a measurement equivalence in our cross-cultural sample [92]. Three models were tested through multigroup CFA, which progressively fixed the number of items and factors (configural invariance—M0), the factor loads (metric invariance—M1) and the intercepts (scalar invariance—M3). The tested model should not present differences in Chi-squared (ΔChi-sqaured), CFI (ΔCFI) < 0.01, McDonald’s NCI (ΔMcDonald’s NCI) < 0.02, RMSEA (ΔCFI) < 0.01 when compared to previous models [93]. Results showed non-differences for factor loadings between countries when configural invariance and the metric invariance models were compared (Δchi-square = 42.23 (Δdf = 3; *p* = 0.00), ΔCFI = 0.01, ΔMc NCI = 0.01, and ΔRMSEA = 0.01). The comparison between the metric invariance and the scalar invariance models also presented non-differences for item intercepts: Δchi-square = 60.49 (Δdf = 3; *p* = 0.00), ΔCFI = 0.01, ΔMc NCI =0.02, and ΔRMSEA = 0.01. It is necessary to mention that Chi-squared index is especially sensitive to the sample size [94], and as well as being an extremely stringent test of invariance for models in CFA [95]. Thus, measurement invariance was supported.

*Objective job insecurity* at the individual level was assessed according to the type of contract, whether temporary or permanent [22]. The following question was used to elicit employee status: “Do you have a permanent contract with this organization?”, with the option to respond either “No” or “Yes”. This variable was coded as a dummy variable: 0 for temporary employment and 1 for permanent employment.

*Job insecurity climate* measurement was obtained through a data aggregation process, as broadly explained in step 2. In sum, individual scores for job insecurity were aggregated at the organizational level. In step 3, we also computed within-organization agreement and between-organization variance to validate this construct at the organizational level.

*Climate strength* of job insecurity was measured as the degree of agreement on perceived job insecurity among employees from a specific organization, as mentioned in step 2. The average deviation index (ADMj) was used for this purpose, as suggested by González-Romá [41]. This index was multiplied by −1, so that higher scores of climate strength reflected high within-organization agreement, and low scores of climate strength reflected low within-organization agreement.

*Job instability in the organization*. The employer of each participant organization responded to a set of questions in relation to job instability in their organization. Specifically, we asked employers to respond to two key questions, the first of which was, “In the past three years, have there been any changes in the number of employees?”, to which there were three possible answers: (1) “Yes, the number of employees increased”; (2) “Yes, the number of employees decreased”; and (3) “No, there were no significant changes in the number of employees”. The second question was, “Which of the following measures have been adopted by your organization in the last three years?”. Employers had to indicate all the measures adopted from the following list: (1) downsizing; (2) record of employment regulation; (3) merger; (4) closure of centers and/or departments; and (5) early retirement plans. Job instability in the organization was codified as a dummy variable based on the combination of these items. High job instability was reflected by reduction in the number of employees and some of the organizational events. Any of the other combinations, for example, reduction descent in the number of employees but no organizational events, or no reduction in the number of employees combined with a presence or lack of organizational events, were coded as no job instability in the organization. This combination was necessary because it is possible for that organizational measures to be adopted without involving a workforce reduction; or an organization might experience a workforce reduction, not as a result of extraordinary measures, but because employees have decided to retire or some temporary contracts have expired. The variable was a dummy coded as 1 for job instability, and 0 for no job stability. The Austrian sample presented six organizations with job instability (16%), while the Spanish sample presented 22 organizations with job instability (35%).

To test step 5, additional measures were used to examine the correlates of job insecurity at multiple levels (function of the construct across levels of analysis). Hence, the construct of job insecurity at multiple levels was related to variables measured at the organizational level, such as nature of organization, and variables measured at the individual level, such as job satisfaction. The following measures were used:

*Nature of organization* was considered as a dummy variable, coded as 1 for private organization, and 0 for public organization.

*Job satisfaction* was assessed with a four-item scale [96] with items such as, “I find enjoyment in my job”, and a response range from 1 (low) to 5 (high). Cronbach’s alpha for this scale was 0.79 in the Spanish sample and 0.70 in the Austrian sample. Regarding measurement invariance, both the comparison between the configural invariance and the metric invariance models showed non-differences for factor loadings (Δchi-square = 18.31 (Δdf = 3; *p* = 0.00), ΔCFI = 0.01, ΔMc NCI = 0.005, and ΔRMSEA = 0.003) and the comparison between the metric invariance and the scalar invariance models indicated non-differences for items intercepts (Δchi-square = 48.94 (Δdf = 3; *p* = 0.00), ΔCFI = 0.03, ΔMc NCI = 0.01, and ΔRMSEA = 0.01). Only, the ΔCFI in the comparison between the metric invariance and the scalar invariance models did not meet the cut-off criteria. However, it was quite close and taking into account that the other indexes supported their invariance, we concluded that measurement invariance was supported. In addition, this variable was also examined at the organizational level, with data aggregated according to multilevel methodology guidelines [80]. The rWG(J) [97] index of agreement and the average deviation index (AD) [98]; Burke, Finkelstein, and Dusig, 1999) were computed to determine whether the individual data were sufficiently homogeneous to be aggregated. The results indicated a good level of agreement among employees within their organizations regarding job satisfaction. The mean and median values of rWG(J) were higher than the cut-off of 0.70 in both samples (James et al., 1993; 1984): Spain, mean = 0.79 and median = 0.85; Austria, mean = 0.77 and median = 0.84. The mean and median AD index values also met the criteria, being lower than the cut-off of 0.83 [98,99]. Specifically, in the Spanish sample, the mean and median AD score was 0.62, and in the Austrian sample, the mean was 0.68 and the median was 0.71.

**Step 3. Psychometric properties of constructs across levels of analysis**

Multilevel constructs require additional evidence about their psychometric properties, such as factor structure, inter-member agreement or reliability of measurements [12]. Measures for objective job insecurity and job instability in organizations were composed of raw scores with only one item, and climate strength was composed using an AD index. Accordingly, the analyses of psychometric properties (e.g., factor structure, internal consistency) were not possible for these last measures.

*Factor structure.* The hierarchical structure in the data of multilevel constructs can involve special requirements in the analysis of their factor structures. Firstly, the independence assumption of the data is violated. Secondly, the nature of the construct can vary across the levels of analysis; therefore, the dimensionality properties and validity may also vary across these levels. In this respect, composition models assume isomorphism, operating practically equally at higher and lower levels of analysis. Finally, the consensus models are considered as valid when there is an adequate level of agreement within the group. In an attempt to deal with these issues, Muthen (1990, 1994) [100] proposed a procedure for performing multilevel confirmatory factor analysis (MCFA), explicitly considering the aggregate nature of the measure. Muthen (1990, 1994) [100] proposed a series of steps to perform MCFA: (1) compute conventional factor analysis; (2) estimation of between variation; (3) estimation of within structure; and (4) estimation of between structure. The final step is the multilevel confirmatory factor analysis itself. We performed MCFA to examine the structure of subjective job insecurity and job insecurity climate at organizational level, using Mplus Version 5.0 [100] in both the Spanish and Austrian samples.

First, a conventional confirmatory factor analysis was computed using the total sample matrix. A one-factor model with paths from the latent construct (subjective job insecurity) to all four items was tested. Overall goodness-of-fit for the models was assessed using a Chi-squared likelihood ratio, normed comparative fit index (CFI) [101], Tucker–Lewis index (TLI) [102], root mean square error of approximation (RMSEA) [103], and the standardized root mean square residual (SRMR). Although the Chi-squared likelihood ratio was significant, the values of the other indexes indicated an adequate fit (see Table 1). Values of CFI and TLI higher than 0.95 were judged to be an acceptable fit in both the Spanish and Austrian samples. RMSEA and SRMR were judged to be appropriate when lower than 0.08 [104]. In both samples, SRMR values met the criteria, though only the Austrian RMSEA was lower than 0.08; the Spanish RMSEA was slightly higher (0.12). The standardized factor loadings for this model are presented in Table 2. All loadings were significant, suggesting that all items adequately reflected the latent construct of job insecurity in both samples.

Secondly, an estimation of within- and between-organization variance was computed through interclass correlation coefficients (ICC) for each item in both samples. Table 2 presents the results. In the Spanish sample, ICCs ranged from 0.33 to 0.44 with an average ICC of 0.39. In the Austrian sample, ICCs ranged from 0.02 to 0.12 with an average ICC of 0.08. Following Dyer [105] procedure, we compared it to the standard ICC(1) values reported by James [106], which tend to range between 0.00 and 0.50, with a median value of 0.12. Similarly, Dedrick and Greenbaum [107] suggested 0.10 as a possible cut-off value. Our ICC values were relatively higher in the Spanish sample whereas, in the Austrian sample, ICC values were around or greater than 0.10, except for item 4. There was, therefore, sufficient between-organization variability to warrant a multilevel methodology in the Spanish sample, but this was less evident in the Austrian sample. Multilevel analyses were computed in both samples, but the results for the Austrian results must be interpreted with caution.

Thirdly, according to Chen [12], it is advisable to compute the within- and between-level models separately prior to estimating the full two-levels model (steps 3 and 4). Within- and between-organization covariance matrices were used to perform these analyses of disaggregate factor structure. In the within-organization covariance matrix, the between-organization variability was removed as the between-organization covariance matrix only reflected this variability. Thus, the pooled within-organization covariance matrix was estimated based on individual deviations around the organization means, while the between-organization covariance matrix was based on the organization means and their deviations around the grand mean. Two single-level confirmatory factor analyses were computed in both Spanish and Austrian samples, one on the sample pooled-within covariance matrix, and the other on the sample between covariance matrix. The goodness-of-fit indices for the model at within and between levels presented an appropriate fit in both Spanish and Austrian samples. The loadings of both analyses differed significantly from zero in both samples. Being very similar in value to the estimates from the final multilevel model, these loadings are not reported in Table 2.

Finally, the multilevel confirmatory factor analysis (MCFA) was performed. The model consisted of two factors, one at the within-organization level (i.e., job insecurity), and the other at the between-organization level (i.e., job insecurity climate). Worthy of note is that the residual variance of item 4 at the between level was fixed to zero to avoid a negative variance estimate. According to Dyer [108], it is often necessary to fix the residual variances at the between level in MFCA when the true between-group residual variance is close to zero, which is the case for item 4 in our Spanish sample. Residual variances were also fixed for the four items in the Austrian sample. The results showed a good fit, similar to the previous analyses (see Table 1). The factor loadings were also significant at both within- and between-levels in both samples. Only item 4 at the within-level in the Austrian sample was not significant. The items loaded more strongly onto the latent factor at between-level (job insecurity climate) than at within-level (job insecurity) in both samples. The factor loadings ranged from 0.59 to 0.80 and from 0.13 to 0.81 at within-level, and from 0.70 to 1.00 and 0.99 to 1.00 at between-level in Spanish and Austrian samples, respectively (Table 2). Higher loadings at the between-organization level compared to those at the within-organization level provided support to the one-factor model across levels, confirming the multilevel model.

*Within-unit agreement.* The focal level for construct validity in composition models is the aggregated level. Two different procedures were used to determine whether the individual data were sufficiently homogeneous to be aggregated: James [97] rWG(J) and the average deviation index [98,99]. The rWG(J) index compares the actual within-unit variance with the expected variance from random responding. The uniform distribution is usually used as recommended by James [109]. In other words, this index considers the extent to which the variability of unit members’ responses presents less dispersion than would be exhibited by chance. The AD index relies on the average standard deviation of items. It does not need explicit modeling of the null random response distribution, which offers an advantage compared to the previous index.

The results showed an appropriate level of within-organization agreement in relation to perceived job insecurity. The mean and median values of rWG(J) were, respectively, 0.70 and 0.83 in the Spanish sample, and 0.81 and 0.86 in the Austrian sample. These indices were higher than the extended cut-off value of 0.70 [97,109]. All AD values were lower than the recommended value of 0.83 for a five-item scale [99]. The mean and median values were, respectively, 0.73 and 0.72 in the Spanish sample, and 0.61 and 0.62 in the Austrian sample.

*Reliability.* The reliability of the measures across levels must be analyzed considering the multiple levels, while the reliability of individual measures and aggregate properties should be examined separately. According to Chen [108], an analysis of psychometric properties must be aligned with the level of analysis. Assuming an adequate degree of agreement for the aggregation, the average item response per organization was computed and the scale reliability was then examined at the aggregate level of analysis (i.e., Cronbach’s alpha).

All alpha values were higher than the commonly accepted cut-off criterion of 0.70 [110]. Specifically, Cronbach’s alpha was 0.89 and 0.80 for subjective job insecurity at individual level, and 0.93 and 0.88 for job insecurity climate at organizational level in the Spanish and Austrian samples, respectively. However, Cronbach’s alpha should not only be interpreted on the basis of its value, but also considering the number of items in scale [111]. Hence, inter-item and item-total correlations were examined. The results were equivalent for the measure of job insecurity at individual and organizational level. All inter-item and item-total correlations were higher than 30 in both samples (see Table 3). Thus, all obtained indices surpassed the minimum value of 0.20 proposed by Streiner [112]. Indeed, all our values exceeded the more stringent cut-off criterion of 0.30 suggested by Cortina [111]. In summary, reliability or internal consistency may be considered as appropriate for subjective job insecurity and job insecurity climate.

**Step 4. Construct variability between units**

As mentioned above, consensus models require an adequate level of inter-member agreement, which justifies the aggregation of ratings within units to a higher-level measure [108]. Inter-member reliability is also necessary, however. Inter-member agreement and reliability provide complementary but different information. Inter-member reliability refers to the extent to which within-unit ratings are proportional (but not identical), whereas within-unit agreement indicates the extent to which within-unit ratings are essentially the same [113].

To test this inter-member reliability, interclass correlations (ICCs) are usually computed. ICC(1) presents the proportion of total variance that can be explained by group membership, in other words, the extent to which within-unit variance is small relative to between-units variance. ICC(2) indicates the degree to which group means can be reliably differentiated. This index reliably detects phenomena among groups that are not detectable by individual-level measures. ICC(1) and ICC(2) values were 0.26 and 0.79, respectively, in the Spanish sample; while in the Austrian sample, ICC(1) and ICC(2) values were 0.11 and 0.57. Both indices presented good reliability in both samples compared to previous research [114].

Finally, Bliese [13] also recommends examining the between-unit variability. Significant between-unit differences support the assumption that the consistency of responses within each unit is greater than the consistency across larger contexts. A one-way analysis of variance (ANOVA) can establish the between-organization differences in perceived job insecurity in both samples. The results (Spain: F(87, 826) = 4.79, *p* < 0.01; Austria: F(48, 452) = 2.33, *p* < 0.01) indicated significant between-organizational differences. Hence, ratings presented greater consistency within each organization than consistency across larger contexts.

**Step 5. Construct function across levels of analysis**

The multi-level construct function involves examination of theoretical models of its relationships with other constructs at different levels of analysis. Such variables can be other multi-level constructs or single-level constructs. It allows us to examine the similarities and dissimilarities in relationships with the construct (job insecurity) at different levels of analysis. In fact, according to Chan (1998) [80], the function of constructs may be similar across levels. Isomorphic constructs with similar meanings across levels probably have homologous relations across levels. The proper alignment of constructs across levels is a key factor in enhancing validity and generalizability [12,115].

**Figure 1:**
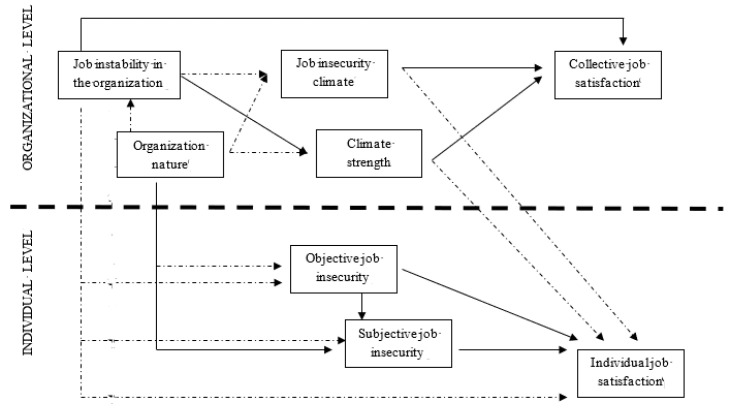
Multi-level model of job insecurity. Note: Continuous lines reflect the significant relationships between variables in one or both samples. Dashed lines represent the non-significant relationships in any sample.

Hence, this model not only involves antecedents, correlates and outcomes of the construct across different levels of analysis, but it also includes relationships between different constructs of job insecurity at different levels. Specifically, Figure 1 displays the proposed multi-level model concerning job insecurity across levels of analysis. This model included job instability in an organization (aggregate properties model), job insecurity climate (direct consensus model) and climate strength (dispersion model) at organizational level, subjective job insecurity and objective job insecurity at individual level, and how these are interrelated. The model further presents antecedent factors and outcomes at individual and organizational level. As organizational antecedents, the model includes organization nature, and presents collective and individual job satisfaction as organizational and individual outcome. These variables were selected because they were theoretically related constructs and previous literature had provided a large empirical support to these relationships at individual level.

*Antecedents: Organization nature*, understood as public vs. private organization, was reported by employers at organizational level and represented an antecedent at the organizational level. Research has associated the public sector with relatively more secure jobs compared to the private sector [116]. For example, the Spanish public sector hires its employees through different types of contract: career civil servant and permanent and temporary employment. The firing of career civil servants is only possible in very extreme cases as they are safeguarded against arbitrary removal from office. Since the labor reform in 2012, the laying off of permanent public employees can be justified for economic, technical, organizational or production reasons. However, the public sector applies alternative organizational measures in preference to permanent workforce downsizing, such as reduction of social benefits or wage freezes. In this respect, Rosenblatt and Mannheim [117,118] reported that public-sector organizations used significantly more non-layoff cutback strategies than private-sector organizations. In contrast, private organizations hire their employees according to their personal merits and the demands of the labor market [116]. Hence, De Witte [19] reported that public employees tend to experience less concern with outright layoff and more concern with losing the job “as they know it” (for example, through reassignment or withdrawal of job prerogatives), compared to private sector employees, who experienced more concern about losing the whole job. Following this evidence, we propose the following hypothesis:

**Hypothesis 1.**  *Private organizations present higher levels of job instability in the organization (H1a), job insecurity climate (H1b), subjective job insecurity (H1c), objective job insecurity (H1d), and weaker job insecurity climate (H1e).*

*Outcomes: Collective and individual job satisfaction.*

Multiple conceptualizations of job insecurity are associated with job satisfaction, according to stress theory [29]. Job satisfaction at the individual level reflects “an internal state that is expressed by affectively and cognitively evaluating an experienced job with some degree of favor or disfavor” [119]. However, Brief (1998) also suggested that this definition could be adapted to represent a construct at a higher level of analysis, such as collective job satisfaction [119]. Hence, Whitman, Van Rooy, and Viswesvaran (2010, p.46) defined collective job satisfaction as a “work unit’s shared internal state that is expressed by affectively and cognitively evaluating shared job experiences with some degree of favor or disfavor” [120]. Collective job satisfaction involves a general predisposition of employees within an organization to collaborate, share, and accept organizational goals [119]. Therefore, collective job satisfaction, understood as the aggregation of individual perceptions of job satisfaction, represents an outcome at the organizational level, whereas individual job satisfaction reflects an outcome at the individual level. Low collective and individual job satisfaction reflect withdrawal behavior in response to job insecurity stressors at both individual (e.g., subjective and objective job insecurity) and organizational levels (e.g., job insecurity climate, strength climate, and job instability in the organization). Empirical studies support the relationship between different conceptualizations of job insecurity and job satisfaction. Regarding job satisfaction at the individual level, most of the research to date has demonstrated a negative relationship between job insecurity and job satisfaction [121], job insecurity climate and job satisfaction [5], and climate strength and job satisfaction [6].

However, to the best of our knowledge, no study has examined the relationship between job insecurity and job satisfaction across or at higher levels. Following the previous research and, according to stress theory [29], we propose that the multiple conceptualizations of job insecurity at the organizational level may also be associated with lower levels of individual job satisfaction:

**Hypothesis 2.**  *High organizational job instability (H2a), job insecurity climate (H2b), and weaker climate strength (H2c) are associated with lower collective job satisfaction.*

**Hypothesis 3.**  *High organizational job instability (H3a), job insecurity climate (H3b), weaker climate strength (H3c), objective job insecurity (H3d), and subjective job insecurity (H3e) are negatively related to individual job satisfaction.*

*Relationship between different constructs of job insecurity at multiple levels*. Organizational job instability, as stated previously, refers to an organizational context in which a downsizing event or practice has taken place, and there is an overlap in the literature on downsizing and job insecurity, as well as a clear linkage in practice [122]. Furthermore, the relationship between downsizing—the core of job instability in this paper—and job insecurity was underpinned by different theoretical frameworks. According to the expectancy theory [123], employees provide services (input) to their organizations, and in turn they expect this to be reciprocated with certain organizational rewards (outcomes). Whitener (1998) highlighted job security as one of these organizational rewards [124]. The social exchange theory [125] also involves implicit rewards in the form of mutual commitment, though no economic payoff is involved. This mutual reciprocity requires an employer to commit to employee job security to win the organizational commitment of employees in return [123,126]. In addition, as mentioned in step 1, job security represents an expectation within the psychological contract, and job threat by downsizing involves a breach of that psychological contract [126].

However, this perception of job insecurity in a downsizing context must not be understood merely as an individual and isolated experience. In a context characterized by workforce reduction, the uncertainty and possibility of job loss is perceived as a real possibility for all employees in that organization. In other words, downsizing can represent a breach of psychological contract for all employees within an organization as well as an imbalance in the exchange relationship between organization and employees, understood as a collective. Hence, it is possible that a general and shared concern of job insecurity can emerge within an organization—a strong job insecurity climate. Following this reasoning, we hypothesize the following:

**Hypothesis 4.**  *Job instability is positively related to subjective job insecurity (H4a), objective job insecurity (H4b), job insecurity climate (H4c), and climate strength (H4d).*

*Objective job insecurity.* As stated in step 1 above, objective job insecurity may be understood as a work stressor, according to stress theory [29], or a breach of the psychological contract [22,33]. In fact, the literature on job insecurity has shown a well-established association between objective and subjective job insecurity, as can be observed in the meta-analyses of Shoss [127] and Sverke [18]. Temporary employees are not part of the core of the organization, and therefore are probably more at risk of losing their jobs; moreover, they are employed by the organization for a limited time according to a temporary contract. Hence, temporary employees experience higher levels of job insecurity compared to permanent employees [18,28]. Accordingly, we hypothesized the following:

**Hypothesis 5.**  *Objective job insecurity is negatively related to job insecurity. Temporary employees perceive higher levels of subjective job insecurity.*

Different methods are used to examine these similarities and dissimilarities in the relationships between multilevel constructs across levels of analysis. On the one hand, multiple random coefficient models were computed to examine the linear relationships suggested in the multilevel model. On the other, multilevel logistic regressions were computed for objective job insecurity and job instability in the organization. These variables were dichotomous (dummy) and required to estimate a generalized mixed-effects model rather than a linear mixed-effects model [128].

Random coefficient models are used to apply different cross-validation principles to examine the extent to which relationships at one level of analysis may generalize to another level of analysis. Other methods, such as WABA or multilevel SEM, are also used to test for similarities and dissimilarities in multilevel theoretical models. However, these methods may be more limiting, especially in relation to aggregated constructs by dispersion or aggregate properties models [12,108]. We also previously computed multilevel prerequisites 86, and group-level properties of outcome variables were computed using ICC(1) values. ICC(1) shows the proportion of the variance in the outcomes that was explained by organizational membership. Indeed, the ICC(1) values obtained were 0.25 and 0.15, for job insecurity and job satisfaction, respectively, in the Spanish sample, and 0.13 and 0.12 in the Austrian sample. In order to examine the intercept variation in outcomes variables, the Chi-square likelihood test was performed, comparing one model with random intercept and another without. Models with random intercepts were significantly higher than those without, in the Spanish sample (job insecurity, −2 log likelihood = 165.69, *p* < 0.01; job satisfaction, 2 log likelihood = 82.21, *p* < 0.01) as well as in the Austrian sample (job insecurity, −2 log likelihood = 20.40, *p* < 0.01; job satisfaction, 2 log likelihood = 14.83, *p* < 0.01). All these indices suggested significant intercept variations in the outcome variables. Overall, it could be concluded that the prerequisites for performing the multilevel analysis were met.

Multilevel logistic regression models were performed to appropriately address organization- and individual-level effects on objective job insecurity and job instability in the organization. As previously mentioned, multilevel logistic regression models can be conducted with dichotomous dependent variables, such as job insecurity and job instability in the organization variables. Hence, the multilevel models are random-intercept models designed to assess the effects of explanatory variables on mean differences in objective job insecurity and job instability in the organization across organizations.

Finally, all continuous variables were centered to reduce risk of multicollinearity [129]. Note that analyses were performed using the random coefficient model program “lme” and the mixed-effects logistic model program “glmer” from R software (version 3.5.3) (R Core Team, 2020) [130].

Results (see Table 4) suggested that organization nature was positively related to subjective job insecurity in the Spanish sample. Spanish private organizations presented higher levels of subjective job insecurity. Furthermore, objective job insecurity was negatively related to subjective job insecurity in both the Spanish and Austrian samples. Temporary employees experienced higher levels of job insecurity compared to permanent employees. Finally, job instability was negatively related to climate strength in the Spanish sample. Organizations with high job instability also presented weaker climates of job insecurity.

Regarding outcomes at higher levels (see Table 5), job instability in an organization was positively related to collective job satisfaction in both the Spanish and Austrian samples. Climate strength was negatively related to collective job satisfaction in both samples, but job insecurity climate was only negatively related to collective job satisfaction in the Austrian sample. In terms of outcomes at the individual level, our results showed a non-significant relationship between the different conceptualizations of job insecurity at higher levels (namely job instability in an organization, job insecurity climate, and climate strength) and individual job satisfaction in both the Spanish and Austrian samples. However, individual subjective job insecurity was negatively related to individual job satisfaction in both samples, while objective job insecurity was only negatively related to individual job satisfaction in the Spanish sample. Spanish permanent employees reported lower levels of individual job satisfaction compared to Spanish temporary employees.

## 3. Discussion

Despite the accumulation and proliferation of studies on job insecurity from different theories and methodologies, research has yet to develop an integrative framework. To do so, this study set out to examine the main constructs related to job insecurity, linking job insecurity with multilevel theory. More specifically, we used advanced multilevel methods aimed at conceptualizing and testing multilevel job insecurity constructs [12]. We directly tested homologous multilevel models and provide evidence for similarities and dissimilarities in models of job insecurity at different levels of analysis in two different European countries—Spain and Austria.

In keeping with the basic principles of multilevel theory [12], our first objective was to define the focal construct at each relevant level of analysis. More specifically, we defined job insecurity at the individual level, understood as a subjective perception and an objective construct we referred to as subjective job insecurity and objective job insecurity, operationalized by temporary employment contracts. Regarding collective constructs, we highlighted job instability in the organization, job insecurity climate, and climate strength.

With the second step, we specified the nature and the structure of the construct at multiple levels of analysis. The structure and nature of job insecurity was explained in terms of the level of theory, the level of measurement and the level of analyses, which coincided at the individual level. However, the structure and nature of the constructs at higher levels were addressed through the composition model [78] and the aggregate properties model [12]. Job insecurity climate was explained in terms of a direct consensus model, climate strength in terms of a dispersion model, and job instability in the organization according to an aggregate properties model.

At the third step, we tested the psychometric properties of the constructs across and/or at different levels of analysis (e.g., the factor structure, inter-member agreement, and reliability of measurements). The results supported the validity and reliability of job insecurity as a global factor structure across different levels in both the Spanish and Austrian samples.

At step 4, the results confirmed that the construct varies between levels of analysis in both the Spanish and the Austrian sample. In other words, between-organizational differences were found concerning levels of job insecurity. Our findings revealed that job insecurity can be an emergent collective property of organizations.

Finally, at step 5, we tested the function of the focal construct across different levels of analysis. According to multilevel research [131], the combined investigation of structure and function is essential for developing concrete and meaningful multilevel constructs. These results shed light on the common antecedents and outcomes at different levels, as well as the relationships among them. Hence, Hypothesis 1, which proposes that organization nature is an organizational antecedent of job insecurity at the individual level (H1c), was partially confirmed in the Spanish sample. In Spain, private organizations present higher levels of subjective job insecurity compared to public organizations. These results were congruent with the previous literature on job insecurity [7,19,116,132].

Hypothesis 2 was supported in the Austrian sample, and partially supported in the Spanish sample. Most of the collective constructs of job insecurity were significantly related to collective job satisfaction. Organizations with job instability (H2a), higher levels of job insecurity climate (H2b) or weaker job insecurity climate (H2c) were associated with higher collective job satisfaction in the Austrian sample. In the Spanish sample, only job instability in an organization (H2a) and climate strength (H2c) were significantly related to collective job satisfaction. These results are congruent with the stress theory of Lazarus and Folkman [29], in which these constructs of job insecurity are perceived as contextual stressors. They also support the literature on job insecurity from a multilevel perspective [5,6]

Hypothesis 3, which proposed the relationships with individual job satisfaction, was also partially confirmed. Subjective job insecurity (H3e) was supported in both the Spanish and the Austrian sample. High levels of subjective job insecurity were associated with lower individual job satisfaction in both Spanish and Austrian employees. These results are congruent with the literature on job insecurity [18,121,127]. Furthermore, objective job insecurity (H3d) was only confirmed in the Spanish sample, and in the opposite direction. In other words, the results indicated that Spanish permanent employees were less satisfied than temporary Spanish employees. Although these results are not congruent with either of our theoretical frameworks, stress theory [29] or psychological contract theory, previous empirical studies have evidenced that temporary employees can show more positive work outcomes than permanent employees [22,133]. Sora [22] suggested that the contextual situation could provide an explanation of these results. A positive association between temporary employment and work outcomes seems more feasible in a prosperous financial context, where job opportunities are numerous and temporary employment might be perceived as less demanding than permanent employment. An alternative explanation could be found in the different types of temporary employment, such as fixed-term, on-call, or independent contractors [134,135]. However, the results were not consistent. Klandermans [135] reported that the outcomes of job insecurity varied according to employment type; whereas Bernhard-Oettel [134] found no differences in employee outcomes as a function of their type of temporary employment. Additional research is therefore necessary to clarify the relationship between objective and subjective job insecurity.

Hypothesis 4, which proposed the relationship between job instability in the organization and job insecurity climate, climate strength, subjective job insecurity and objective job insecurity, was only supported in relation to climate strength in the Spanish sample. Job instability in the organization was negatively related to climate strength; hence, Spanish organizations with high job instability presented weaker climates of job insecurity. These results are congruent with the literature on climate strength, which reports that weaker climates are more detrimental and stressful for employees [40,75,76]. It seems plausible that downsizing events within organizations promote higher levels of uncertainty about job permanency, hence we find higher dispersion and differences in perceived job insecurity according to the employee’s job and personal characteristics.

Hypothesis 5 was supported in both samples. Temporary employees reported higher levels of subjective job insecurity compared to permanent employees. This is congruent with the literature on job insecurity [18,127], which states that temporary employment does not represent the core of an organization’s workforce. Temporary employees are perceived as more expendable and with more potential for job loss compared to permanent workers, hence temporary employees experience higher levels of job insecurity compared to permanent workers.

Taken together, this work contributes to job insecurity research by examining and validating the nature of job insecurity construct at higher levels. Job insecurity phenomenon must be conceptualized not only as an individual perception, but also as a collective experience that is shared among employees within an organization. Analogous to individual job insecurity, job insecurity at higher levels can be conceptualized from objective and subjective perspectives. Therefore, job instability in organizations can represent an objective and collective job insecurity within organization, whereas job insecurity climate would reflect a collective and subjective phenomenon. Finally, it is critical to highlight that job insecurity at higher levels must be understood as content and strength.

Job insecurity at higher levels was presented as a contextual stressor. Despite theoretical models of job insecurity at multiple levels have proposed similar antecedents and outcomes across levels [1,3,5,6,57], this work showed both similarities and dissimilarities in the models of job insecurity at different levels. The results suggested that the antecedents and outcomes of job insecurity at the individual level were not necessarily the same for job insecurity at the organizational level; that is, job insecurity at the organizational level was not isomorphic to job insecurity at the individual level. As Chen [108] suggested, “the assumption of homology is too often assumed and too rarely tested”. Thus, our results reveal a possible need to rethink the theoretical assumption of homology in multilevel models of job insecurity. Findings from research on job insecurity at individual levels cannot necessarily be generalized to job insecurity at higher levels, and vice versa.

This study also evidenced the differences among European countries by finding variety in the relationships between multilevel job insecurity constructs in function of country. Whereas the associations between job insecurity at multiple levels and job satisfaction at multiple levels were similar in Spain and Austria, the effect of the organizations and countries’ characteristics on employees varied in function of country. More specifically, the results showed non-significant relationships between organizations and countries’ characteristics in Austria compared to Spain, where significant associations between organization nature and subjective job insecurity, job instability in organization and climate strength, and objective job insecurity and job satisfaction were found. A potential explanation of these results could underpin labor market characteristics. First, the Spanish labor context seems to be more segmented between permanent and temporary employment and private versus public organizations in terms of employment protection compared to the Austrian labor market. Hence, it is possible to find significant associations in the Spanish sample, and not in the Austrian one. More specifically, the Spanish labor market seems to be more instable and uncertain for employees. It presents higher rates of temporary employment (25.1%), and of unemployment (19.4%) compared to Austrian temporary employment (9%) and unemployment rates (5.3%) from 2011 to 2021 [90]. Second, the Spanish law is stricter for dismissal procedures for permanent employees compared to Austrian law. These procedures are more restrictive in Spain than in Austria when considering, for example, notification procedures that involves the obligation to provide a written statement with the reasons for dismissal or severance pay [136]. Organization nature also seems to provide higher employment protection in Spain than in Austria. In Austria, the population expressed that it is wrong to assume that a public law status automatically confers more protection and more disciplinary rights against dismissal than a labor law status. In contrast, the Spanish population expressed that agreed working conditions are more respected by public employers than by private ones, and job security is taken for granted [137]. Third, industrial relations are also an important factor in the employment protection. Unions’ primary concern is to secure jobs for all workers and to reduce the number of workers in precarious jobs [138]; thus, a successful union representation can promote job stability and security. Therefore, as De Cuyper [7] suggested, we take union density and collective bargaining coverage as indicative of the power of unions to influence personnel staffing strategies. Data shows that Austrian unions have more impact compared with Spanish unions, because the proportion of unionized workers in Austria was at 27.3% compared to 15.1% percent in Spain from 2011 to 2021 [90]. Finally, in Spain, the economic crisis was very pronounced at the time the data were collected. The economic prospect, widely discussed in the mass media, was even worse. This negative prospect may have increased concern about job loss and its consequences, in contrast to Austria, a country which was less affected by the economic crisis. Only a few studies on job insecurity have adopted a cross-cultural approach [139,140,141]. In fact, König [140] state that “little is known about how people in different countries react to job insecurity” (p. 150), and argue for more context-sensitive research on job insecurity.

In conclusion, in order to advance in the understanding of job insecurity phenomenon, future research must consider models of job insecurity at multiples levels with cross-cultural research designs.

Limitations. Despite the contribution of this study, it also presents a number of limitations. Causal inferences cannot be drawn from the study because of its cross-sectional research design: future longitudinal research will be needed to infer these causal relationships. In addition, most of our measures were self-reported; common method variance is therefore possible. Additional measures would be advisable in future research.

### 3.1. Theoretical and Practical Implications

Up to now, the growing research on job insecurity has examined this work stressor at multiple levels, but without the necessary basis or substantive framework. This study proposed this integrative framework that includes the main conceptualizations of job insecurity; in other words, it proposed an integration of the different constructs of job insecurity at individual and higher levels by adopting a multilevel approach. This study clarified and validated the nature of these constructs in relation to the different levels, and it evidenced that though these constructs may share similarities, they also manifest differences in different ways. Therefore, job instability in organizations and job insecurity climate included uncertainty about job continuity, but they differ in their nature. Job instability in organizations represents a more objective threat to job continuity, whereas job insecurity climate reflects a collective subjective perception, similarly to the differentiation between subjective job insecurity and objective job insecurity, understood as type of contract, at the individual level. In this vein, another important contribution was the difference between job insecurity climate and subjective job insecurity. Most of the literature has studied these constructs from the isomorphism assumption. However, this study evidenced that job insecurity climate is closely linked to its lower-level namesake (job insecurity), but they differ in important ways, as is evidenced on employees’ outcomes. In sum, this study underpinned the understanding nature of constructs at higher levels and its validity in order to pursue multilevel research and theory related to job insecurity construct. This study also involved important practical implications. Practicians and managers must take care about job stability within their organizations from different aspects. Organizations must prevent the individual perception of job insecurity or their employees’ concern about job lost, but also they should facilitate supportive climates for employees. This individual work stressor can also be presented at collective levels. So, if the organization is able to prevent this perception from being shared by most employees by using a clear and informative policy of employment, it will reduce the negative effects of job insecurity at multiple levels on employees’ outcomes. In sum, this study offered the multilevel validity of job insecurity construct through an integrative framework in order to advance in the area of job insecurity theory and practice.

### 3.2. Future Research

Our findings suggest several important areas for future research. Firstly, the mixed results in the Spanish and Austrian sample indicate potential differences based on culture and labor markets. Future research could examine these multilevel job insecurity constructs in multiple countries to explain this variability. Secondly, the results indicate that a valuable area of research may be a further examination of similarities and differences in perceived job insecurity, and antecedents and consequences of its conceptualization. Thirdly, a potential boundary for the generalizability of these results is the type of work units that we examined. Sudstrom (1999) and Chen (2002) [12,142] suggested that organizational phenomena may vary according to teams. In this respect, future research could focus on examining these job insecurity constructs at other levels of analysis, such as departments or teams. In this vein, other higher levels could be also considered to examine the collective phenomenon of job insecurity. For example, it has already suggested that countries with specific cultural factors and labor market characteristics may affect levels of job insecurity. Future research could examine if it is possible that collective job insecurity could emerge at higher levels than the organizational level, such as country level. Fourth, we used a consensus composition model to obtain the measure of job insecurity climate through an aggregation process of individual subjective job insecurity data, and climate strength was measured using an AD index. These measures did not allow us to examine the relationships between these constructs at higher levels, and job insecurity at lower levels. Future research could focus on the development of new measures based on shift-referent models that facilitate understanding of the relationships between these job insecurity constructs [3].

## 4. Conclusions

This work broadens the focus of job insecurity conceptualization by validating the nature of this construct at multiple levels. It conceptualized job insecurity at the individual level—understood as subjective and objective job insecurity—and at the organizational level, understood as job instability in an organization, job insecurity climate, and climate strength. By testing a homologous multilevel model of job insecurity, this study has evidenced both similarities and dissimilarities in the models of job insecurity. Accordingly, it is necessary to reconsider the theoretical assumptions of homology in multilevel models of job insecurity. Results and theoretical assumptions from research on individual job insecurity must not be generalized to collective job insecurity, and vice versa. In conclusion, we hope that this research encourages researchers to examinate job insecurity from a multilevel perspective, taking into account if it differs or remains the same across levels of analysis, in order to advance in the understanding of this work stressor and its consequences for employees and organizations.

## Figures and Tables

**Table 1:** Model fit for a priori single- and multilevel models.

	χ^2^	*df*	*p*	CFI	TLI	RMSEA	SRMR
Spanish sample							
Step 1: Total	965.29	6	0.00	0.99	0.96	0.12	0.02
Step 3: Within	308.03	6	0.00	0.99	0.97	0.06	0.02
Step 4: Between	238.43	6	0.00	0.97	0.91	0.25	0.02
Step 5: Multilevel	466.35	12	0.00	0.95	0.88	0.10	W = 0.01; B = 0.04
Austrian sample							
Step 1: Total	295.32	6	0.00	1.00	1.01	0.00	0.00
Step 3: Within	1224	6	0.97	1.00	1.00	0.00	0.00
Step 4: Between	105.64	6	0.00	1.00	1.05	0.00	0.01
Step 5: Multilevel	201.03	12	0.00	0.97	0.96	0.04	W = 0.00; B = 0.37

df = degrees of freedom, CFI = comparative fit index, TLI = Tucker-Lewis index, RMSEA = root mean square error of approximation, SRMR = standardized root mean square residual. W = within-organization portion of the model, B = between-organization portion of the model.

**Table 2:** Standardized factor loadings from a priori single- and multilevel models and intraclass correlations (ICC) by scale item.

**Item**	Standardized loadings in Spanish sample			
	**Step 1: Total**	**Step 5: Within**	**Step 5: Between**	**Step 2: ICC**
Item 1	0.87	0.80	0.93	0.43
Item 2	0.81	0.72	0.98	0.35
Item 3	0.89	0.80	1.00	0.44
Item 4	0.61	0.59	0.70	0.33
	Standardized loadings in Austrian sample			
Item 1	0.72	0.70	0.99	0.09
Item 2	0.53	0.47	1.00	0.12
Item 3	0.83	0.81	0.99	0.11
Item 4	0.14	0.13	0.99	0.02

**Table 3:** Inter-item and item-total correlations for job insecurity at multiple levels.

	*Spanish sample at individual level Spanish sample at organizational level*									
	Inter-item correlations				Item-total correlation					Item-total correlation
	Item 1	Item 2	Item 3	Item 4		Item 1	Item 2	Item 3	Item 4	
Item 1	-				0.83	-				0.90
Item 2	0.73	-			0.78	0.83	-			0.86
Item 3	0.83	0.77	-		0.84	0.89	0.87	-		0.90
Item 4	0.62	0.58	0.59	-	0.65	0.71	0.66	0.66		0.71
*Austrian sample at individual level Austrian sample at organizational level*										
	Inter-item correlations				Item-total correlation					Item-total correlation
	Item 1	Item 2	Item 3	Item 4		Item 1	Item 2	Item 3	Item 4	
Item 1	-				0.65	-				0.77
Item 2	0.44	-			0.53	0.68	-			0.75
Item 3	0.65	0.50	-		0.72	0.77	0.71	-		0.83
Item 4	0.55	0.43	0.62	-	0.62	0.62	0.65	0.73	-	0.74

**Table 4:** Summary of analyses predicting job insecurity at multiple levels.

	Subjective Job Insecurity			Objective Job Insecurity			Job Insecurity Climate			Climate Strength			Job Instability in the Organization		
	Spanish sample														
	PE	SE	*p*	PE	SE	*p*	PE	SE	*p*	PE	SE	*p*	PE	SE	*p*
Intercept	2.48	0.20	0.00	1.42	0.50	0.00	0.55	0.24	0.02	0.36	0.06	0.00	−19.38	6.14	0.00
Objective job insecurity	−0.82	0.10	0.00												
Organization nature	0.46	0.21	0.03	0.84	0.56	0.13	−0.08	0.26	0.74	−0.04	0.07	0.52	0.85	6.62	0.89
Job instability in org.	0.10	0.17	0.53	−0.63	0.47	0.18	−0.21	0.29	0.47	−0.21	0.09	0.00			
	Austrian sample														
	PE	SE	*p*	PE	SE	*p*	PE	SE	*p*	PE	SE	*p*	PE	SE	*p*
Intercept	1.90	0.13	0.00	2.06	0.37	0.00	1.50	0.11	0.00	−0.57	0.08	0.00	−58.30	45.68	0.20
Objective job insecurity	−0.42	0.09	0.00												
Organization nature	0.11	0.12	0.34	−0.72	0.41	0.08	0.04	0.12	0.71	0.14	0.09	0.12	−0.32	6.68	0.96
Job instability in org.	0.21	0.14	0.14	−0.50	0.47	0.29	0.28	0.15	0.08	−0.06	0.11	0.57			

Note: PE, parameter estimate. SE, standard error. Objective job insecurity (0 temporary; 1 permanent). Organization nature (0 public organization; 1 private organization). Analyses are random coefficient models for all outcomes, except for objective job insecurity and job instability, which were multilevel logistic regressions.

**Table 5:** Random coefficient models predicting collective and individual job satisfaction.

	Collective Job Satisfaction			Job Satisfaction		
	PE	SE	*p*	PE	SE	*p*
	Spanish sample					
Intercept	0.26	0.18	0.15	4.56	0.16	0.00
Job instability in the organization	0.90	0.36	0.01	−0.00	0.09	0.99
Job insecurity climate	−0.10	0.24	0.65	0.01	0.08	0.94
Climate strength	−4.01	0.23	0.00	0.21	0.17	0.23
Subjective job insecurity				−0.27	0.03	0.00
Objective job insecurity				−0.23	0.08	0.00
	Austrian sample					
Intercept	1.68	0.20	0.00	4.22	0.20	0.00
Job instability in the organization	3.94	0.34	0.00	−0.02	0.12	0.86
Job insecurity climate	−2.10	0.45	0.00	−0.32	0.20	0.12
Climate strength	−2.81	0.07	0.00	0.03	0.30	0.93
Subjective job insecurity				−0.25	0.05	0.00
Objective job insecurity				0.01	0.09	0.94

Note: PE, parameter estimate. SE, standard error. Objective job insecurity (0 temporary; 1 permanent).

## Data Availability

The data that support the findings of this study are available on request from the corresponding author. The data are not publicly available due to privacy or ethical restrictions. The data reported in this article were collected as part of a larger data collection. Other studies have been published with this dataset, but the research objectives and hypothesis examined in the present article have not been examined in any previous or current articles, or to the best of our knowledge in any papers that will be under review soon.
